# Comparative proteomic analysis of cucumber powdery mildew resistance between a single-segment substitution line and its recurrent parent

**DOI:** 10.1038/s41438-019-0198-3

**Published:** 2019-10-15

**Authors:** Xuewen Xu, Xueli Liu, Yali Yan, Wei Wang, Kiros Gebretsadik, Xiaohua Qi, Qiang Xu, Xuehao Chen

**Affiliations:** School of Horticulture and Plant Protection, Yangzhou University, 225009 Yangzhou, Jiangsu China

**Keywords:** Protein sequencing, Biotic

## Abstract

Powdery mildew (PM) is considered a major cause of yield losses and reduced quality in cucumber worldwide, but the molecular basis of PM resistance remains poorly understood. A segment substitution line, namely, SSL508-28, was developed with dominant PM resistance in the genetic background of PM-susceptible cucumber inbred line D8. The substituted segment contains 860 genes. An iTRAQ-based comparative proteomic technology was used to map the proteomes of PM-inoculated and untreated (control) D8 and SSL508-28. The number of differentially regulated proteins (DRPs) in SSL508-28 was almost three times higher than that in D8. Fourteen DRPs were located in the substituted segment interval. Comparative gene expression analysis revealed that nodulin-related protein 1 (NRP1) may be a good candidate for PM resistance. Gene Ontology enrichment analysis showed that DRPs functioning in tetrapyrrole biosynthetic process, sulfur metabolic process and cell redox homeostasis were specifically enriched in the resistant line SSL508-28. DRPs categorized in the KEGG term photosynthesis increased in both lines upon PM infection, suggesting that the strategies used by cucumber may be different from those used by other crops to react to PM attacks at the initial stage. The measurement of hydrogen peroxide and superoxide anion production and net photosynthetic rate were consistent with the changes in protein abundance, suggesting that the proteomic results were reliable. There was a poor correlation between DRPs measured by iTRAQ and the corresponding gene expression changes measured by RNA-seq with the same experimental design. Taken together, these findings improve the understanding of the molecular mechanisms underlying the response of cucumber to PM infection.

## Introduction

Cucumber (*Cucumis sativus* L., 2*n* = 2x = 14) is an important vegetable crop worldwide. Powdery mildew (PM) is a destructive fungal disease that is globally distributed and that affects a wide range of agricultural and horticultural crops, including cucumber^1^. *Podosphaera xanthii* has been identified as the main cause of PM on cucumber^2^. The application of fungicides is the conventional method of managing PM in most cucumber production areas^3^. In response to growing consumer concerns for pesticide residues, alternative/additional strategies for disease control are required^4^. The development of PM-resistant cultivars is the most desirable strategy to control PM^3^.

Accumulating knowledge regarding the molecular mechanisms of host defenses is a prerequisite for crop improvement^5^. In work involving forward genetic approaches, quantitative trait loci (QTLs) related to cucumber PM resistance have been identified in all cucumber chromosomes (Chr) except for Chr6, supporting the hypothesis that PM resistance in cucumber is likely due to the combinatorial effects of several genes^6–10^. The step from mapping to the identification of the gene through map-based cloning has been a challenge due to the quantitative nature of the resistant trait. Physiological or morphological plasticity allows higher plants to adapt to undesirable abiotic or biotic stress, but these adaptations require sophisticated regulatory networks to simultaneously modulate the expression of multiple genes and proteins^11^. Genomic and postgenomic technologies, including transcriptomics, proteomics, and mass spectrometry, are therefore necessary for understanding the mechanism by which plants alter their signaling and physiological responses to beneficial vs. pathogenic microbes^12^. Comparative transcriptomic analysis of cucumber PM-resistant parent S1003 and susceptible near-isogenic line (NIL; *Pm5.1*) at 12 h after PM inoculation showed that the underlying resistance might be correlated with plant cell-wall thickening^13^. However, PM resistance in S1003 is recessively inherited, which is not convenient for use in cucumber breeding^3,9^. The characterization of dominant resistance is required and will help build a more complete picture of PM resistance in cucumber.

In a previous study, a segment substitution line (SSL), namely, SSL508-28, was developed using dominant PM resistance introgression from Jin5-508 in the genetic background of PM-susceptible D8^3,5^. Whole-genome resequencing revealed that only a single 6.8 Mb segment on Chr5 (designated *Pm5.1* hereafter) from the donor was introgressed into SSL508-28^5^. Comparative RNA-seq-based transcriptome analysis of the leaves of SSL508-28 and D8 48 h after PM inoculation revealed a complex regulatory network mediated by *Pm5.1* that included several signal transducers or regulators, the salicylic acid signaling pathway and cell-wall modifications^5^. However, the detection of changes in transcript abundances does not necessarily indicate that the same change occurs in the expression of the corresponding proteins because of the existence of extensive post-transcriptional regulation or alternative splicing^14^. Proteomics is a high-throughput approach to address gene functions that cannot be identified by sequencing and is the most direct way of obtaining a coherent picture of the role of a gene^15^. In the current study, we employed an iTRAQ-based quantitative proteomic approach to reveal the proteins and pathways underlying PM resistance in SSL508-28 and PM susceptibility in the recurrent parent D8. The results of this study will improve the understanding of the molecular mechanisms underlying the response of cucumber to PM infection triggered by the *Pm5.1* locus.

## Results

### PM resistance phenotype

**Fig. 1:**
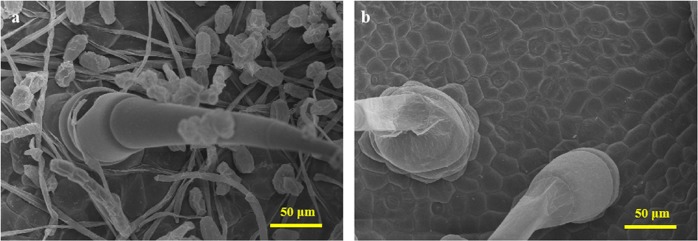
Microscopic observations of leaves 48 h after powdery mildew inoculation. **a** D8 leaf; **b** SSL508-28 leaf

To understand the phenotypic difference between the resistant and susceptible lines, we observed the extent of PM growth on the leaf surface by scanning electron microscopy (SEM). We observed dense PM hyphae on the surface of D8 leaves (Fig. 1a), whereas no conidia were detected on the surface of SSL508-28 (Fig. 1b). The result was consistent with higher resistance in the resistant line SSL508-28 than in the susceptible parent D8.

### Inventory of leaf proteins identified by iTRAQ

**Fig. 2:**
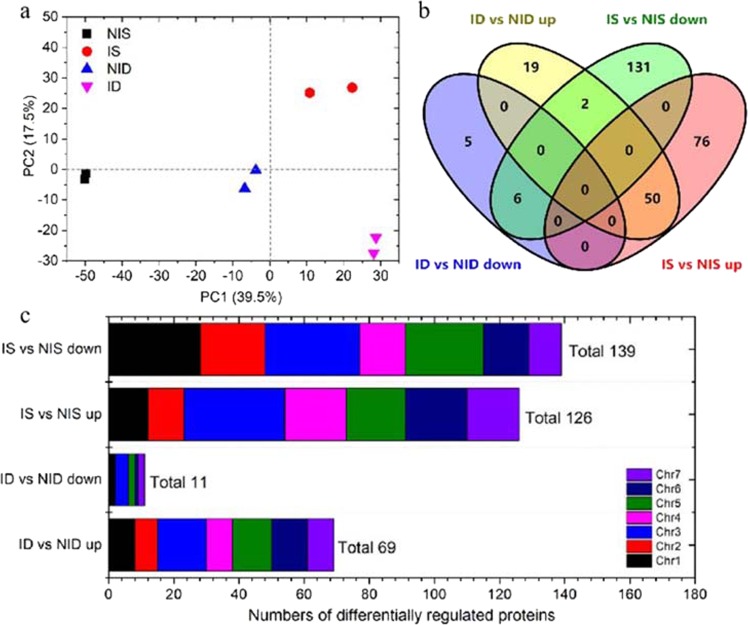
Expression patterns of differentially regulated proteins (DRPs). **a** Principal component analysis (PCA) of the iTRAQ data. Each point in the PCA graph represents the whole-protein profile of one biological replicate. Numbers in parentheses on the axes represent the percentage of total variance explained by each principal component. **b** Venn-diagram of the distribution of DRPs. **c** Distribution of DRPs on each chromosome (Chr) of cucumber. ID: PM-inoculated D8 leaves; NID: noninoculated D8 control leaves; IS: PM-inoculated SSL508-28 leaves; NIS: noninoculated SSL508-28 control leaves. “ID vs. NID up” represents those DRPs with higher expression in D8 leaves at 48 h after infection with PM when compared with mock-inoculated D8 leaves; “ID vs. NID down” represents those DRPs with lower expression in D8 leaves at 48 h after infection with PM when compared with mock-inoculated D8 leaves. “IS vs. NIS up” represents those DRPs with higher expression in SSL508-28 leaves at 48 h after infection with PM when compared with mock-inoculated SSL508-28 leaves; “IS vs. NIS down” represents those DRPs with lower expression in SSL508-28 leaves at 48 h after infection with PM when compared with mock-inoculated SSL508-28 leaves

To elucidate the host defense mechanisms underlying the difference in resistance between the two genotypes upon PM infection, we applied an iTRAQ proteomic technology to leaves of seedlings harvested 48 h after PM inoculation. Using the protein pilot software, we matched 70,571 spectra to known spectra and identified 22,157 distinct peptides and 6966 proteins in the eight samples. We assessed the reproducibility of the iTRAQ data using principal component analysis. The results showed clear separation between the SSL508-28 and D8 data, and between the control and PM-inoculated samples (Fig. 2a). The eight samples were divided into four major groups, with the two replicates in each group.

Pairwise comparisons of proteins with *P*-values < 0.05 and fold-changes > 1.5 or < 0.67 in abundance were regarded as differentially regulated proteins (DRPs). We identified a total of 80 DRPs, including 69 upregulated and 11 downregulated proteins, by comparing the PM-infected D8 (ID) against the noninoculated D8 (NID) control (Supplementary Table S2). In contrast, we obtained 265 DRPs, including 126 upregulated and 139 downregulated proteins, by comparing the inoculated SSL508-28 (IS) against the noninoculated SSL508-28 (NIS) (Supplementary Table S3). We identified 207 DRPs (131 increased and 76 decreased in abundance) only in SSL508-28 and 24 DRPs (19 increased and 5 decreased in abundance) only in D8. Two proteins (Csa3G252490 and Csa4G664300) were upregulated in ID vs. NID but downregulated in IS vs. NIS (Fig. 2b). The DRPs were located on all seven cucumber chromosomes (Fig. 2c).

### Classification of DRPs identified by iTRAQ

**Fig. 3:**
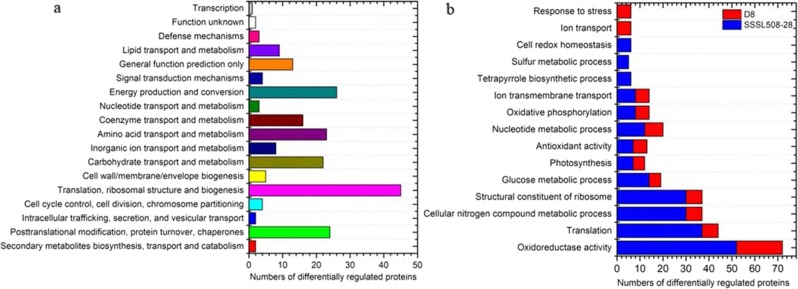
Functional annotation of differentially regulated proteins (DRPs). **a** COG classification of DRPs. **b** Comparison of selected significantly enriched GO terms. The red bar represents DRPs identified in D8 leaves at 48 h after infection with PM compared with mock-inoculated leaves; the blue bar represents DRPs identified in SS508-28 leaves at 48 h after infection with PM when compared with mock-inoculated leaves

Using the COG database, we classified the identified DRPs into 18 categories. The largest category was translation, ribosomal structure, and biogenesis (45 DRPs), followed by energy production and conversion (26 DRPs), post-translational modification, protein turnover, chaperones (24 DRPs), amino acid transport and metabolism (23 DRPs), and carbohydrate transport and metabolism (22 DRPs) (Fig. 3a). We used Gene Ontology (GO) annotation to determine the significantly enriched GO functional groups for the DRPs. The DRPs identified in IS vs. NIS and ID vs. NID were annotated through GO enrichment analysis using the online agriGO tool (http://bioinfo.cau.edu.cn/agriGO). Enrichment analysis of GO functions showed that 40 GO terms were shared between SSL508-28 and D8, including translation (GO:0006412), oxidoreductase activity (GO:0016491), antioxidant activity (GO:0016209), and photosynthesis (GO:0015979). We found that 73 GO terms, including tetrapyrrole biosynthetic process (GO:0033014), sulfur metabolic process (GO:0006790), and cell redox homeostasis (GO:0045454), were specifically enriched in the resistant line SSL508-28. However, only 18 GO terms were specifically enriched in the susceptible parent D8, including ion transport (GO:0006811) and response to stress (GO:0006950) (Fig. 3b).

To investigate the functional consequences of the DRPs associated with PM infection, we used KEGG pathway mapping. The results showed that PM infection affected carbon metabolism, photosynthesis, ribosomes, oxidative phosphorylation, and sulfur metabolism in both lines. The KEGG terms cyanoamino acid metabolism, pyruvate metabolism, fatty acid metabolism, and RNA degradation were highly enriched in the DRPs that were unique to the IS vs. NIS pair (resistant line SSL508-28). The KEGG terms peroxisome, sulfur metabolism, and thiamine metabolism were selectively enriched in DRPs in the ID vs. NID pair (susceptible parent D8) (Table 1).

**Table 1:** KEGG pathway enrichment analysis of differentially regulated proteins (DRPs). The web-based program KOBAS 3.0 (http://kobas.cbi.pku.edu.cn/anno_iden.php) was used to analyze the enrichment

#	KEGG term	Pathway ID	DRPs in SSL508-28	*P*-value	DRPs in D8	*P*-value
1	Carbon metabolism	ath01200	37	4.67E-30	11	3.69E-10
2	Photosynthesis	ath00195	8	1.60E-06	7	4.62E-09
3	Ribosome	ath03010	35	2.13E-23	8	1.14E-05
4	Oxidative phosphorylation	ath00190	11	9.49E-07	6	9.06E-06
5	RNA degradation	ath03018	4	0.025	-	-
6	Cyanoamino acid metabolism	ath00460	3	0.022	-	-
7	Pyruvate metabolism	ath00620	6	2.43E-04	-	-
8	Fatty acid metabolism	ath00061	3	0.009	-	-
9	Peroxisome	ath04146	-	-	2	0.027
10	Sulfur metabolism	ath00920	-	-	2	0.007
11	Thiamine metabolism	ath00730	-	-	1	0.034

### Comparative analysis of protein abundance and gene expression levels

**Fig. 4:**
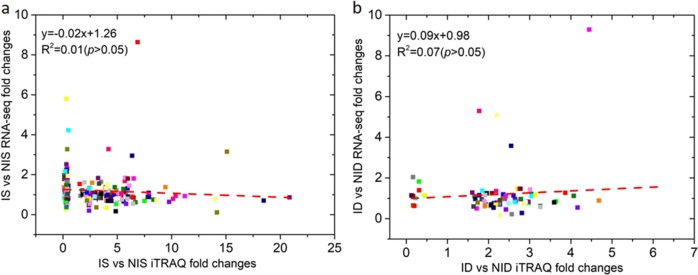
Correlation analysis of protein abundance changes (*x*-axis) measured by iTRAQ and the corresponding gene expression changes measured by RNA-seq (*y*-axis). **a** Comparison of IS vs. NIS, comparing PM-inoculated SSL508-28 (IS) leaves with noninoculated SSL508-28 (NIS) control leaves 48 h after PM inoculation; **b** Comparison of ID vs. NID, comparing PM-inoculated D8 (ID) leaves and noninoculated D8 (NID) control leaves 48 h after PM inoculation

We examined the correlation between protein expression and gene expression to investigate whether changes in messenger RNA (mRNA) expression led to changes in protein abundance after PM inoculation. We determined mRNA expression levels corresponding to the DRPs using the RNA-seq-based transcriptome dataset with the same experimental design^5^. Pearson’s correlation coefficients (PCC values) of the DRPs and mRNA pairs were 0.01 and 0.07 for IS vs. NIS and ID vs. NID, respectively (Fig. 4). There was no significant correlation in either group at the 0.05 level (two-tailed). Thus, there was a poor correlation between transcript levels and protein abundance. The discrepancy between the two omics levels has been reported previously and may be attributed to post-translational regulation or a technical limitation of the iTRAQ approach that makes comparisons difficult^16,17^.

### DRPs located on the substituted segment

The annotation of the 6.8 Mb introgressed fragment predicted 860 genes. The predicted functions of the genes and associated information is shown Supplementay Table S1. Among the genes, 50 were annotated as transcription factors (TFs) based on their assigned protein families, accounting for nearly 6% of the genes located in the fragment. Among the TFs, C_2_H_2_ (*n* = 8), MYB (6) and bHLH (5) were the most frequently identified groups. In addition, 27 genes were predicted to encode protein kinases, including 18 genes encoding receptor-like protein kinases, 5 leucine-rich repeat receptor protein kinases and 4 serine/threonine protein kinases. However, no *mildew resistance locus o* gene was found. The DRPs located within the introgressed substitution segment (*Pm5.1*) were considered possible candidates for important proteins determining the difference in resistance between the parent and the resistant line. A total of four upregulated DRPs (Csa5G524750, Csa5G576620, Csa5G602750, and Csa5G606550) within the introgressed segment were found in the ID vs. NID pair (Table 2). In contrast, 11 DRPs, including five upregulated (Csa5G524830, Csa5G568810, Csa5G580620, Csa5G589260, and Csa5G606550) and six downregulated (Csa5G495940, Csa5G568310, Csa5G588730, Csa5G589930, Csa5G589950, and Csa5G590210) proteins were identified in the IS vs. NIS pair (Table 2). Among these 14 DRPs, the only DRP shared between the two genotypes was nodulin-related protein 1 (NRP1, Csa5G606550), which was upregulated in both SSL508-28 and D8, but the protein induction was higher in SSL508-28.

**Table 2:** Differentially regulated proteins located in the introgressed segment

#	Protein ID	IS vs. NIS	ID vs. NID	Functional annotation
1	Csa5G524830	2.49 ± 1.02	-	Protease Do-like 1
2	Csa5G568810	2.95 ± 0.81	-	HMG-Y-related protein A-like
3	Csa5G580620	4.12 ± 2.05	-	Acid alpha galactosidase 1
4	Csa5G589260	4.69 ± 1.78	-	Cysteine synthase
5	Csa5G606550	15.10 ± 2.32	4.45 ± 0.51	Nodulin-related protein 1
6	Csa5G495940	0.35 ± 0.09	-	Elongation factor 1-alpha-like
7	Csa5G568310	0.34 ± 0.19	-	Phosphoglucomutase
8	Csa5G588730	0.26 ± 0.10	-	60S ribosomal protein L5
9	Csa5G589930	0.12 ± 0.07	-	Photosystem II 47 kDa protein
10	Csa5G589950	0.27 ± 0.06	-	60S ribosomal protein L17-2-like
11	Csa5G590210	0.39 ± 0.06	-	Signal recognition particle 72 kDa protein-like
12	Csa5G524750	-	3.64 ± 0.17	50S ribosomal protein L12
13	Csa5G576620	-	2.35 ± 0.36	ATPase beta subunit
14	Csa5G602750	-	1.85 ± 0.04	Major latex protein-like protein 423-like

ID vs. NID represents the comparison in protein abundance between PM-inoculated D8 leaves (ID) and noninoculated D8 (NID) control leaves at 48 h after inoculation; IS vs. NIS represents the comparison between PM-inoculated SSL508-28 (IS) leaves and noninoculated SSL508-28 (NIS) control leaves at 48 h after PM inoculation

Mean values ± SD

**Fig. 5:**
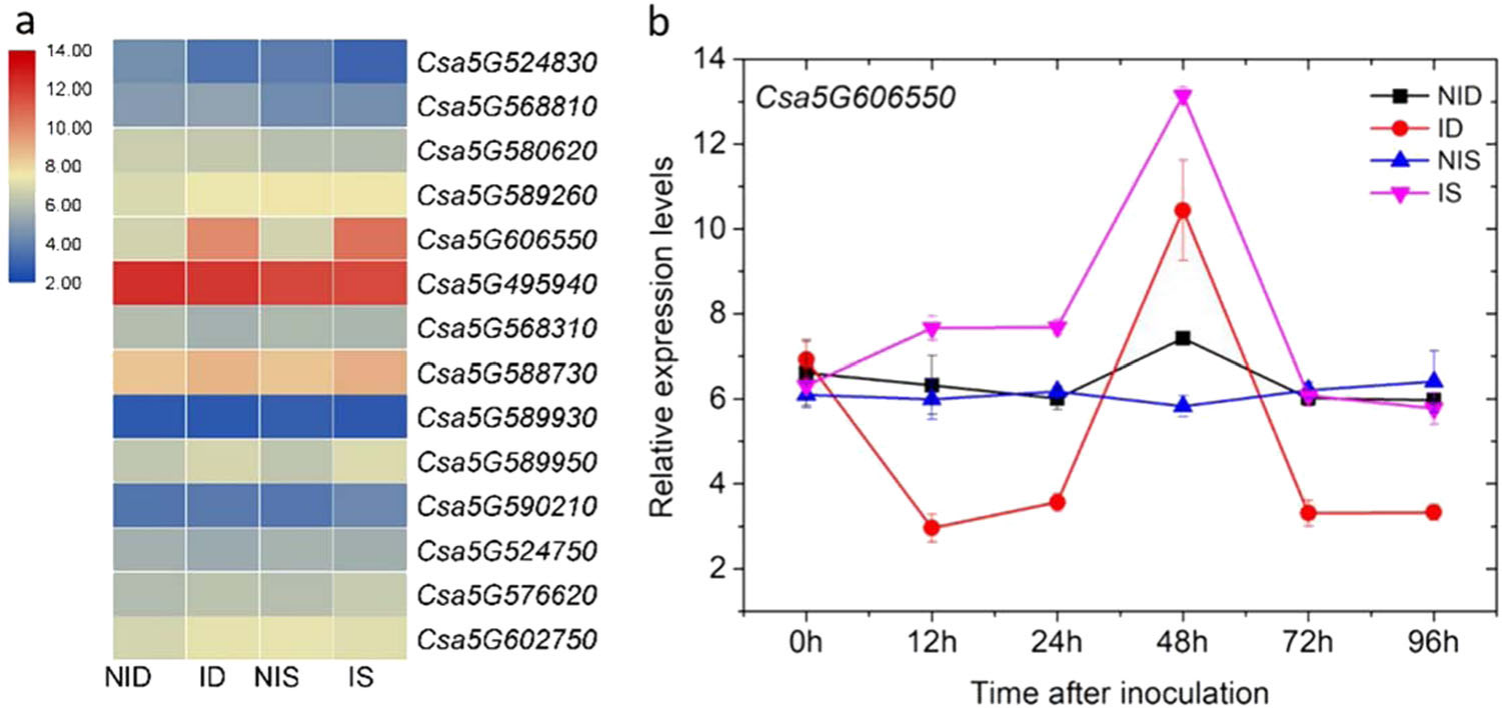
Expression analysis of selected genes located on the substituted segment in D8 and SSL508-28. Data are the means of three replicates ± SD. **a** qRT-PCR analysis of the 14 differentially regulated proteins at 48 h after powdery mildew (PM) inoculation. Genes highly or weakly expressed in the leaves are colored red and blue, respectively. The heat map was generated using TBtools v0.6644449. **b** Expression dynamics of *Csa5G606550* in D8 and SSL508-27 after PM inoculation. NID: noninoculated control D8 leaves; ID: PM-inoculated D8 leaves; NIS: noninoculated control SSL508-28 leaves; IS: PM-inoculated SSL508-28 leaves

We evaluated the expression of these 14 DRPs at the transcriptional level by quantitative reverse transcription PCR (qRT-PCR) analysis. Of the 14 genes, three (*Csa5G524830*, *NRP1* and *Csa5G588730*) were significantly changed in expression in both D8 and SSL508-28 at 48 h after PM inoculation, while only *NRP1* showed similar trends to the results of iTRAQ analysis (Fig. 5a and Table 2). We further investigated the expression dynamics of *NRP1* in D8 and SSL508-27 at 0, 12, 24, 48, and 96 h after inoculation with the PM pathogen (Fig. 5b). The relative expression of *NRP1* was significantly higher in IS than in the NIS leaves at 12, 24, and 48 h after treatment; the peak expression occurred at 48 h after inoculation. In contrast, no consistent trends in expression levels were found in D8 upon PM infection, with lower expression at 12, 24, 72, and 96 h, but higher expression at 48 h (Fig. 5b). The increased expression in PM-inoculated leaves of SSL508-28 (IS) suggested an important role for *NRP1* in PM defense.

### Histochemical detection of endogenous reactive oxygen species accumulation

**Fig. 6:**
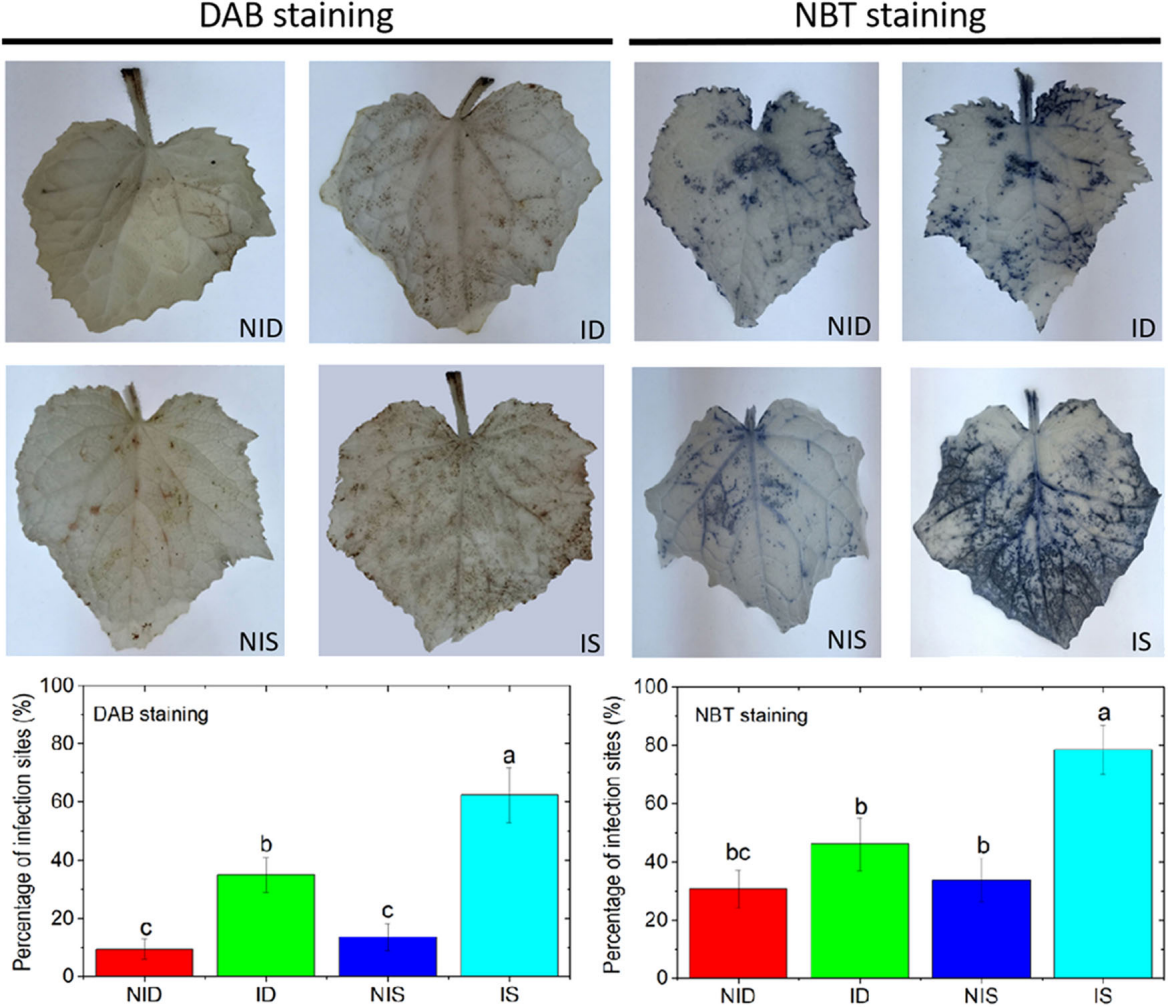
Hydrogen peroxide (DAB staining) and superoxide ion (NBT staining) production in D8 or SSL508-28 leaves inoculated with powdery mildew (PM). Images obtained after DAB and NBT staining were quantified using ImageJ software. Data are the means ± SE of three independent experiments with three biological replicates in each experiment. Bars with different letters denote significant differences at *P* < 0.05. ID: PM-inoculated D8 leaves at 48 h after inoculation; NID: noninoculated D8 control leaves; IS: PM-inoculated SSL508-28 leaves at 48 h after PM inoculation; NIS: noninoculated SSL508-28 control leaves

GO enrichment analysis showed that cell redox homeostasis was among the classes of DRPs enriched in SSL508-28 (Fig. 3b). These DRPs included five thioredoxins (Trxs, Csa1G651650, Csa3G104920, Csa6G343710, Csa2G346600, and Csa2G345990) and one thioredoxin reductase (Csa4G169490). Cellular redox homeostasis, determined by the interplay between the accumulation and scavenging of reactive oxygen species (ROS), plays a positive role in the adaptive response by acting as a signal to activate defense responses^18^. To determine whether PM inoculation affected ROS homeostasis, we used 3,3′-diaminobenzidine tetrahydrochloride (DAB) and nitroblue tetrazolium (NBT) staining to detect the respective amounts of hydrogen peroxide (H_2_O_2_) and superoxide anion (O_2_^−^) (two major kinds of ROS) in SSL508-28 and D8 leaves 48 h after PM inoculation (Fig. 6). Images obtained after DAB and NBT staining were quantified using ImageJ software. DAB precipitation significantly increased in both lines after PM inoculation but was higher in SSL508-28 than in D8. In the O_2_^−^ evaluation, the amount of NBT staining in SSL508-28 after PM inoculation was significantly higher than that in the uninoculated controls, but PM inoculation did not affect the O_2_^−^ levels in D8 leaves (Fig. 6).

### Influence of PM infection on leaf net photosynthetic rate

**Fig. 7:**
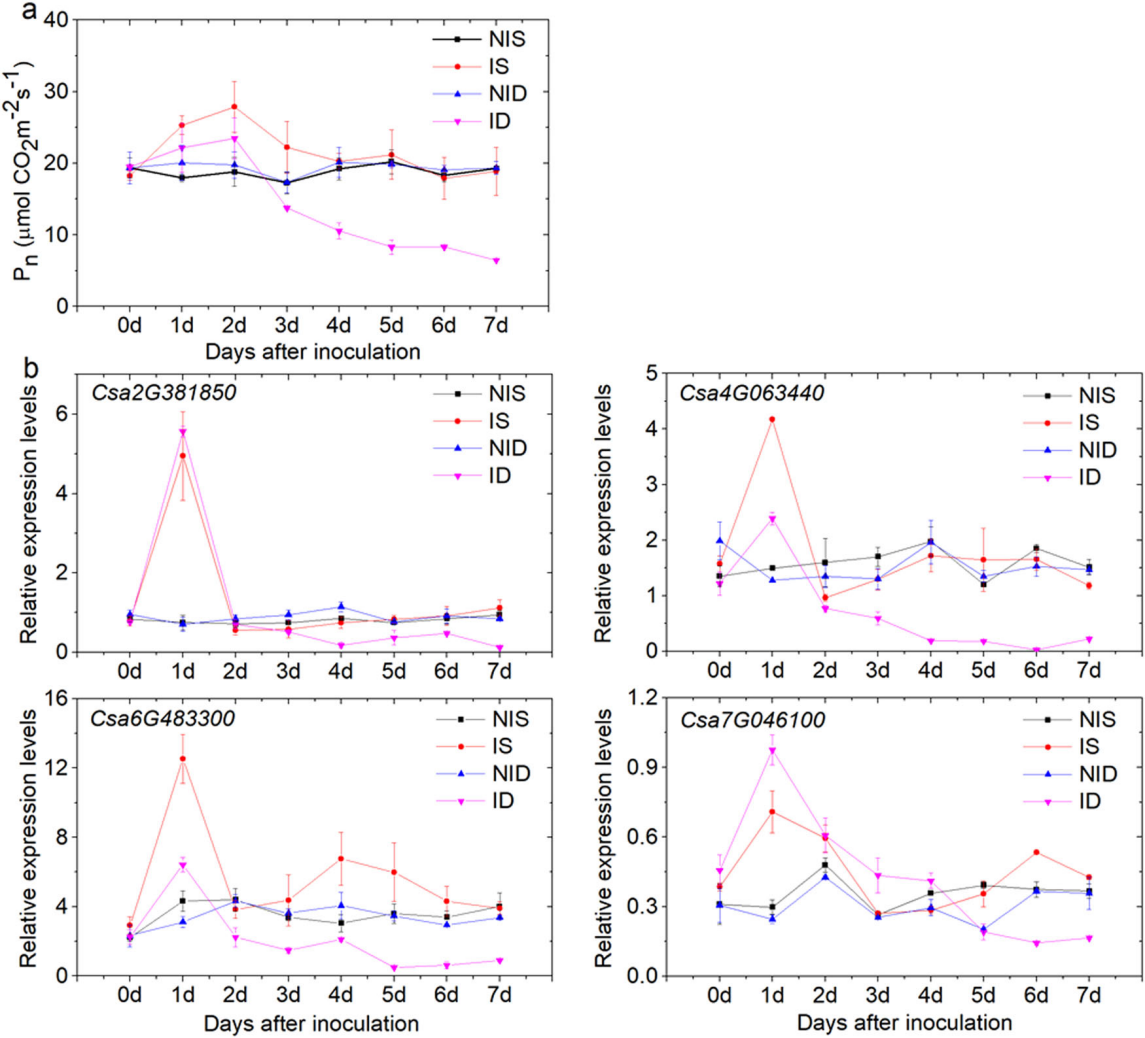
Effect of powdery mildew (PM) infection on net photosynthetic rate (*P*_n_) and photosynthesis-related gene expression in cucumber seedlings. Data are the means ± SE of three independent experiments with three biological replicates for each experiment. NID: noninoculated D8 control leaves; ID: PM-inoculated D8 leaves; NIS: noninoculated SSL508-28 control leaves; IS: PM-inoculated SSL508-28 leaves

Eight DRPs in SSL508-28 and seven DRPs in D8 were assigned to the KEGG term “photosynthesis” (Table 3). Among them, Csa2G381850.1, Csa4G063440.1, Csa6G483300.1, and Csa7G046100.1 were significantly accumulated in both lines, but all at higher levels in SSL508-28 than in D8 (Table 3). Considering the observed proteomic responses, we measured the photosynthetic rate in the leaves of D8 and SSL508-28 at 0, 1 day, 2 day, 3 day, 4 day, 5 day, 6 day, and 7 day after inoculation of the PM pathogen to determine whether the PM infection influenced photosynthesis in the plants. The leaf net photosynthetic rate (*P*_n_) was upregulated in both lines at 1 and 2 day after PM inoculation but was significantly higher in PM-inoculated SSL508-28 than in PM-inoculated D8. In contrast, the *P*_n_ in PM-inoculated SSL508-28 was similar to that in the uninoculated controls after 3 days of treatment, whereas it consistently decreased in PM-inoculated D8 at all three time points (Fig. 7). To further examine the relationship between photosynthesis and the PM infection response, we investigated the expression patterns of *Csa2G381850*, *Csa4G063440*, *Csa6G483300*, and *Csa7G046100*. The correlation analysis revealed that the expression levels of the four genes were positively correlated with *P*_n_ in PM-inoculated D8 leaves (Supplementary Table S4). However, no obvious significant associations between the expression levels of these four genes and the *P*_n_ were identified in the PM-inoculated SSL508-28 leaves or noninoculated controls (Supplementary Table S4).

**Table 3:** Differentially regulated proteins involved in photosynthesis

#	Protein ID	IS vs. NIS	ID vs. NID	Functional annotation
1	Csa2G381850	13.98 ± 1.39	6.20 ± 2.00	ATP synthase subunit b’
2	Csa4G063440	8.60 ± 1.55	1.91 ± 0.45	Oxygen-evolving enhancer protein 2
3	Csa6G483300	5.81 ± 1.30	3.26 ± 0.31	Photosystem I reaction center subunit N
4	Csa7G046100	18.48 ± 2.36	3.60 ± 0.28	Cytochrome b6-f complex iron-sulfur subunit
5	Csa1G066480	9.78 ± 1.38	-	Oxygen-evolving enhancer protein 3-2
6	Csa3G119660	14.17 ± 4.47	-	psbQ-like protein 1
7	Csa6G016970	6.46 ± 1.26	-	ATP synthase delta chain
8	Csa7G047350	3.01 ± 0.31	-	Photosystem II repair protein
9	Csa4G064020	-	2.66 ± 0.49	Photosystem II 10 kDa polypeptide
10	Csa6G488340	-	2.26 ± 0.85	Oxygen-evolving enhancer protein 1
11	Csa6G016970	-	3.26 ± 0.12	ATP synthase delta chain

ID vs. NID represents the comparison of the protein abundance between PM-inoculated D8 leaves (ID) and noninoculated D8 (NID) control leaves at 48 h after inoculation; IS vs. NIS represents the comparison of PM-inoculated SSL508-28 (IS) leaves and noninoculated SSL508-28 (NIS) control leaves at 48 h after PM inoculation. Mean values ± SD

## Discussion

In this study, an SSL carrying a PM resistance locus from Jin5-508 (PM-resistant donor) was used to investigate cucumber PM resistance. This approach allowed the precise detection of defense proteins because of the high level of uniformity in the genetic background of the SSL and its parent, except for the substituted segment from the donor. Microscopic observations showed high numbers of PM hyphae on the surface of D8 (susceptible parent) leaves, while no conidia were detected on the surface of SSL508-28 (resistant SSL) leaves (Fig. 1). In our previous study^5^, through comparative RNA-seq-based transcriptome analysis of the leaves of SSL508-28 and D8 at 48 h after PM inoculation, we identified eight candidate genes that might participate in PM resistance, including two tandemly arrayed genes encoding receptor protein kinases (*Csa5G600370* and *Csa5G600380*), two transcription factors (*Csa5G569350* and *Csa5G606310*), two genes encoding remorin proteins (*Csa5G606540* and *Csa5G606730*), one gene encoding a P-type ATPase (*Csa5G604040*), and one gene encoding a 70 kDa heat shock protein (Csa5G512930)^5^. Here, through iTRAQ-based proteome analysis, we found that 14 DRPs were located in the substituted segment (Table 2). Unfortunately, no common candidate was detected by both RNA-seq and iTRAQ methods, suggesting that the translational and post-translational regulatory mechanisms responsible for PM resistance afforded by *Pm5.1* differ significantly^19^. Therefore, the use of iTRAQ-based comparative proteomic analyses can help comprehensively elucidate molecular processes and help identify additional candidate proteins.

Although the DRP that is the genetic basis for resistance remains unclear, comparative analyses have created a small pool of candidates for further study. Among the DRPs, of particular interest was NRP1 (Csa5G606550). Evidence showed that the NPR1 gene (*At2g03440*) was induced and provided a protective defense response when *Arabidopsis* leaves infiltrated with *Pseudomonas syringae* pv. *tomato* DC3000^20^ and when roots were colonized by *Pseudomonas thivervalensis*^21^ and that this gene plays a negative role in the regulation of the ABA synthesis pathway. In the current study, we found that NRP1 was induced in both SSL508-28 and D8 but was induced to a greater extent in SSL508-28 than in D8 (Table 2). The expression dynamics of *NPR1* (*Csa5G606550*) in SSL508-28 and D8 further indicated the importance of the gene in responding to PM inoculation (Fig. 5). Thus, it can be inferred that the upregulation of NRP1 might act as a positive regulator and contribute to cucumber PM resistance by regulating ABA synthesis.

The candidate proteins include an elongation factor 1-alpha (EF1α, Csa5G495940), which has been identified as a regulator of DNA replication/repair protein networks and has an important function in apoptosis^22^. Decreased EF1α expression results in resistance to apoptosis^23^. This has led to the speculation that a change in EF1α expression in SSL508-28 may be one of the factors that modulates the rate of apoptosis induced by infection with the PM fungus. In addition, major latex-like protein (MLP, Csa5G602750) was an important candidate. MLPs belong to the pathogenesis-related ten-like protein family, which contains proteins with similar three-dimensional structures but low sequence similarity^24^. The functions of MLPs in cucumber disease resistance are controversial. Chen and Dai^25^ found that the expression of *Gossypium hirsutum Gh-MLP* was rapidly induced within 10 min and was maintained at high levels after inoculation with *Verticillium dahliae* strain Vd991, but *Gh-MLP* transgenic plants exhibited similar wilt symptoms to the control. Wang et al.^26^ found that expression of the MLP gene (GenBank accession *JF261109*) and MLP-like gene (GenBank accession *JF261110*) decreased after *V. dahliae* infection in wilt-resistant cotton. In our study, we found that MLP was specifically highly accumulated in susceptible D8 following PM inoculation.

Comparative proteomic analysis of SSL508-28 and D8 in this study also provided several potential insights into the host defense mechanisms mediated by *Pm5.1*. First, the resistance triggered by *Pm5.1* might be closely related to cell redox homeostasis. Histochemical analysis with DAB and NBT staining revealed that ROS accumulated in both resistant and susceptible genotypes but were higher in resistant SSL508-28 than in susceptible D8 in the initial stage (48 h) of PM infection. The accumulation of ROS molecules might represent an early event in the induction of genes/proteins involved in defense signaling and oxidative metabolism to trigger defense responses that limit pathogen expansion^27^. However, excess ROS production in the cell can also trigger programmed cell death. Trxs are key actors that modulate ROS scavenging; functional loss of Trx results in altered ROS levels^28^. Among the five differentially regulated Trxs, Csa1G651650 and Csa6G343710 accumulated in both lines, suggesting that plants might perceive changes in ROS balance and common detoxification mechanisms in PM-infected leaves. The elevated expression of Csa2G346600 and Csa6G343710 in the resistant genotype SSL508-28 might aid in the consumption of the excess harmful ROS generated during the PM infection. Catalase (EC1.11.1.6) is found in eukaryotic peroxisomes, where it converts H_2_O_2_ to water and oxygen^29^. In this study, we found that catalase (Csa4G658600) was highly induced in D8, which may help explain the lower H_2_O_2_ levels detected in PM-infected D8, as indicated by the lower intensity of DAB staining (Fig. 6).

The second potential mechanism raised by this study is that PM resistance mediated by *Pm5.1* might be related to photosynthesis. It is known that pathogen invasion results in a reduction in the rates of plant photosynthesis, such as in potato with *Phytophthora*^30^ and citrus with *Xanthomonas citri* pv. *citri*^31^. Li et al.^32^ found that proteins involved in photosynthesis were mostly downregulated in the compatible interaction of wheat with the PM pathogen *Blumeria graminis* f. sp. *tritici*. Eight DRPs in SSL508-28 and seven DRPs in D8 were assigned into the KEGG term photosynthesis. In contrast, all of the DRPs evaluated in this study accumulated during PM invasion, which was supported by the *P*_n_ measurement (Fig. 7a). These results suggested that the strategies employed by cucumber are different from those employed by wheat to react to PM attacks. As discussed by Rojas et al.^33^ although the light reactions of photosynthesis produce chloroplastic ROS, which can trigger defense responses, the decrease in photosynthesis was counterintuitive, and no experimental evidence is currently available to explain this occurrence. Rinaldi et al.^34^ found that genes related to photosynthesis were upregulated in polar leaves at 48 h after infection with the rust fungus *Melampsora larici-populina*. Photosynthesis might be induced and thus provides energy, carbon skeletons and reducing equivalents required to support a subsequent defense response^35^. Interestingly, among the photosynthesis-related DRPs, ATP synthase subunit b’ (Csa2G381850), the oxygen-evolving complex of photosystem II (Csa4G063440), photosystem I reaction center subunit N (Csa6G483300), ATP synthase delta chain (Csa6G016970) and cytochrome b6-f complex iron-sulfur subunit (Csa7G046100) were shared between SSL508-28 and D8. However, all four DRPs were induced to a greater extent in SSL508-28 than in D8 (Table 3). As expected, *P*_n_ in PM-inoculated SSL508-28 leaves was higher than that in PM-inoculated D8 leaves within 3 days after PM infection (Fig. 7). These results suggest that photosynthesis is an important component of the cucumber response to early stages of PM infection. It is thus tempting to speculate that greater efficiency of photosynthesis in SSL508-28 can supply additional energy that supports a subsequent defense response. The significant correlation between *P*_n_ and the expression levels of the four commonly regulated genes in D8, but not in SSL508-28, confirms that further invasion of PM pathogens will affect leaf photosynthesis (Supplementary Table S4).

The third potential mechanism underlying resistance is that the PM resistance mediated by *Pm5.1* might trigger disease/defense-related proteins. The plant cell wall is mainly composed of polysaccharides and represents the first mechanical barrier against fungal pathogens; the wall needs to be broken for successful invasion^36^. Polygalacturonases (PGs) are the most important enzymes secreted by phytopathogenic fungi at the very early stages of the infection process; they degrade the plant cell-wall through hydrolysis of polygalacturonan (a cell-wall component) into oligosaccharides, thus providing nutrition for the fungus and supporting further infection^37^. To counteract the activity of PGs, plants deploy cell wall-binding polygalacturonase-inhibiting proteins (PGIPs) to limit fungal invasion by inhibiting the activity of PGs^38^. Transgenic *Arabidopsis* plants overexpressing *PGIP1* or *PGIP2* showed increased resistance to *Fusarium graminearum*, while silencing of *PGIP1* led to reduced resistance to *Botrytis cinerea*^39^. In field experiments, Wang et al.^40^ found that transgenic rice overexpressing *OsPGIP1* showed improved resistance against *Rhizoctonia solani*. In this study, iTRAQ analysis showed that the leucine-rich repeat (LRR) protein PGIP1 (Csa4G154320) accumulated in both lines but was almost four-fold higher in SSL508-28 (6.88 ± 0.44) than in D8 (1.77 ± 0.11), suggesting a crucial role for PGIP1 in cucumber against infection by the PM pathogen (Supplementary Tables S2 and S3). In addition to PGIP1, our iTRAQ study also found that another differentially regulated LRR domain, containing protein DNA damage repair/toleration 100 (DRT100, Csa4G290740.1), was specifically accumulated in SSL508-28 (Supplementary Table S3). The upregulation of *DRT* (T44979) was detected in the leaf tissue of canola infected with *Alternaria brassicicola*^41^. Fujimori et al.^42^ found that transgenic *Arabidopsis* overexpressing *Vitis vinifera DRT100-L* showed a reduced frequency of DNA single-strand breaks compared with the wild type. It is tempting to speculate that increased DRT100 in SSL508-28 might be responsible for the repair of DNA damage caused by PM pathogen attack.

In addition to the DRPs discussed above, we identified 34 and 7 differentially regulated ribosomal proteins (RPs) in SSL508-28 and D8, respectively. Although most RPs are thought to be part of core housekeeping proteins involved in translation and show constitutive expression, some of them were found to be differentially regulated upon pathogen infection^43^. Tobacco expressing a truncated *RP L3* from yeast showed resistance to *Fusarium* mycotoxin DON^44^. Both *RP L12-* and *RP L19*-silenced *Nicotiana benthamiana* plants showed varying extents of delay in initiation of the hypersensitive response against infection by *P. syringae* pv. *glycinea* or *X. campestris* pv. *vesicatoria* pathogen^45^. Gong et al.^46^ reported that *V. dahliae* infections induced the expression of cotton *RP L18*, and knockdown plants became more susceptible to the disease. These findings confirmed the role of RPs in plant defense. However, in our study, most of the identified PRs exhibited decreased expression (28 in SSL508-28 and 5 in D8) after PM inoculation (Supplementary Tables S2 and S3), suggesting the divergence of the roles of the RPs family in cucumber PM defense. The precise roles of these RPs in PM defense require further investigation.

## Materials and methods

### Plant materials and PM inoculation

Seedlings of the cucumber single-substitution line SSL508-28 and parent line D8 were grown in a growth chamber with 14/10 h and at 28/22 °C (day/night). Conidia of PM were harvested from naturally infected leaves of D8 in the greenhouse. The spore suspension was diluted to 10^6^ spores mL^−1^, and 0.01% Tween-20 was added. Healthy seedlings at the two-leaf stage were artificially inoculated by spraying the preprepared spore suspension evenly on the leaf surface. Seedlings treated with sterile water (also containing 0.01% Tween-20) were used as a mock inoculation control. The leaves were harvested 48 h after inoculation. Samples were frozen immediately in liquid nitrogen and stored at −80 °C until analysis.

### Protein extraction and iTRAQ analysis

Leaves were ground into powder in liquid nitrogen, transferred to chilled acetone containing 10% (v/v) trichloroacetic acid, and incubated for 1 h at −20 °C. After centrifugation at 15,000 × *g* at 4 °C for 15 min, the protein pellet was vacuum-dried and redissolved in lysis buffer (8 M urea, 2 M thiourea, 4% CHAPS, 40 mM Tris/HCl, 10 mM dithiothreitol, pH 8.5) containing 2 mM EDTA and 1 mM phenylmethylsulfonyl fluoride (PMSF). After sonication for 10 min, the homogenate was centrifuged at 25,000 × *g* at 4 °C for 20 min to prepare the microsomal fraction. The supernatants were transferred to a new tube, mixed with 5 × volumes of prechilled acetone and incubated for 2 h at −20 °C. After centrifugation, the resulting protein pellet was vacuum-dried and dissolved in 0.25 mL tetraethyl ammonium bicarbonate (500 mM, pH 8.5). The protein concentration was quantified by NanoDrop (Thermo Scientific, Waltham, MN, USA). Protein integrity was verified by polyacrylamide gel electrophoresis.

iTRAQ analysis was performed by Shanghai OE Biotech Co., Ltd. (Shanghai, China). Two biological replicates for each sample were prepared. Modified trypsin (3.3 µg; Promega, Madison, WI, USA) was added to 100 µg of protein, and then the mixture was digested for 24 h at 37 °C. The solvent was removed by speed vacuum. The peptides were labeled using an iTRAQ 8-plex labeling kit (Applied Biosystems, Thermo Scientific) following the manufacturer’s protocol. Peptides of noninoculated D8 (control) were labeled with iTRAQ tags 113 and 114, while PM-inoculated D8 were labeled with iTRAQ tags 115 and 116. Peptides of noninoculated SSL508-28 (control) were labeled with iTRAQ tags 117 and 118, while PM-inoculated SSL508-28 were labeled with iTRAQ tags 119 and 121. The labeling reactions were incubated at room temperature for 2 h. The peptides were purified by SCX column (250 × 4.6 mm, 5 µm, 100 Å; Phenomenex Luna, Torrance, CA, USA) on a high-performance liquid chromatography system (Shimadzu, Kyoto, Japan). The retained peptides were eluted with buffer A (10 mM KH_2_PO_4_ in 25% acetonitrile, pH 2.8) and buffer B (10 mM KH_2_PO_4_, 2 M KCl in 25% acetonitrile, pH 3.0) and then pooled into 20 fractions, desalted with a Strata X C18 column (Phenomenex) and vacuum-dried. All flow rates were 1 mL min^–1^.

Each peptide fraction was immersed in 5% acetonitrile containing 0.1% formic acid, and the final peptide concentration was adjusted to 0.5 µg µL^−1^. Fractions (10 µL) were loaded onto an Eksigent nanoLC-Ultra™ 2D system (AB Sciex, Foster City, CA, USA) equipped with a C18 column (100 µm × 3 cm, C18, 3 µm, 150 Å). Peptides were eluted with a linear gradient of 5–30% buffer B (0.1% formic acid in 95% acetonitrile) over 70 min at a flowrate of 300 nL min^−1^. The collected peptides were subjected to mass spectrometry using a TripleTOF 5600 Analyzer (AB Sciex, Foster City, CA, USA) using the following settings: ion spray voltage, 2.5 kV; curtain gas, 30 psi; ion source gas, 5 psi; and interface heater temperature, 150 °C. All raw mass spectrometry data were deposited in the iProX Consortium (an official member of ProteomeXchange Consortium) with the dataset identifier IPX0001253001.

### Identification of peptides and proteins

The raw files were analyzed using Protein Pilot software v. 5.0 (AB Sciex) with the Paragon algorithm against the cucumber 9930 genome assembly v. 2.0 (http://www.icugi.org). The following search parameters were used: (1) sample type: iTRAQ8plex (peptide labeled); (2) Cys alkylation: iodoacetamide; (3) digestion: trypsin; (4) instrument: TripleTOF 5600; (5) search effort: rapid. The results were filtered based on a false-discovery rate of no >1%. Protein identification was supported by at least two unique peptides, and the unused ProtScore was higher than 1.3.

### SEM imaging

For SEM, PM-inoculated SSL508-28 and D8 leaves were collected, cut into 5 × 5-mm pieces and fixed overnight in 4% glutaraldehyde. Samples were mounted on aluminum stubs and sputter-coated with Au-Pd. The specimens were then viewed by field emission SEM (S-4800, Hitachi, Japan) at an accelerating voltage of 10 kV.

### DAB and NBT staining

Detection of H_2_O_2_ was performed by DAB staining^47^. O_2_ was detected by in situ histochemical staining using NBT^48^. PM-infected leaf segments were immersed and vacuum-infiltrated with staining solution containing 0.4% DAB or 0.1% NBT in 10 mM MES (pH 6.4) for 4 h. Stained leaves were bleached in boiling (100 °C) acetic acid:glycerol:ethanol (1:1:3 v/v) for 5 min and stored in 96% ethanol until photographed^49^.

### Determination of *P*_n_ and chlorophyll concentration

The second true leaf was used for *P*_**n**_ and chlorophyll concentration determination. *P*_n_ was measured with a portable photosynthesis system (LI-6400, LI-COR, Lincoln, USA) at a photon flux density of 500 µmol m^−2^ s^−1^. Fresh leaves (0.5 g) were ground and then extracted with chilled 80% acetone until the tissue was completely bleached. The suspension was centrifuged at 6000 × *g* at 4 °C for 10 min. The chlorophyll concentration in the supernatant was determined by spectrophotometry (Spectrum SP-752, Shanghai, China).

### qRT-PCR analysis

Total RNA was isolated using a MiniBEST Universal RNA Extraction Kit (Takara, China), dissolved in water-DEPC and adjusted to a final concentration of 1 mg mL^−1^ using NanoDrop™ One (Thermo Scientific). RNA was reverse-transcribed using a Takara PrimeScript^®^ RT reagent kit with a genomic DNA eraser following the manufacturer’s instructions. qRT-PCR was performed using a Tiangen RealMasterMix (SYBR Green) kit (Beijing, China). PCR cycling was performed using an iQ5 multicolor real-time PCR detection system (Bio-Rad, Hercules, CA, USA) with 20-µL samples. Primers (Supplementary Table S5) used in the qRT-PCR analysis were designed using the online software Primer3 (v. 0.4.0, http://bioinfo.ut.ee/primer3-0.4.0/). The cucumber *βuactin* gene (GenBank AB010922) was used as an internal control. A correlation analysis between *P*_n_ and gene expression levels was performed with SAS 9.0 software.

## Supplementary information

Table S1

Table S2

Table S3

Table S4

Table S5
